# Real-world insights into incidence and risk factors of thyroid dysfunction following immune checkpoint inhibitor therapy

**DOI:** 10.3389/fimmu.2026.1882182

**Published:** 2026-08-07

**Authors:** Lingyan Zhou, Qinran Long, Ying Zhang, Mei Zhan

**Affiliations:** 1 Department of Pharmacy, West China Hospital, Sichuan University, Chengdu, China; 2 West China School of Pharmacy, Sichuan University, Chengdu, China; 3 Department of Pharmacy, Institute of Metabolic Diseases and Pharmacotherapy, West China Hospital, Sichuan University, Chengdu, China

**Keywords:** hypothyroidism, immune checkpoint inhibitors, immune-related adverse events, thyroid dysfunction, thyrotoxicosis

## Abstract

**Background:**

Real-world findings regarding the risk factors for immune checkpoint inhibitor (ICI) -related conditions remain scarce and show a lack of consistency.

**Research design and methods:**

We conducted a retrospective study of patients who treated with ICIs between October 2014 and June 2023. Patient follow-up was extended until death or July 4, 2025. Logistic regression was used to investigate the associations between clinical characteristics and thyroid dysfunction after ICI initiation.

**Results:**

After excluding 411 patients with missing key data and 1,342 with pre-existing thyroid dysfunction, 6,857 patients were included in the final cohort. ICI-related thyroid dysfunction was identified in 1,342 cases (19.57% overall), with 582 subclinical hypothyroidism, 155 overt hypothyroidism, 521 central hypothyroidis, and 84 thyrotoxicosis. Logistic regression identified subtype-specific risk factors. For subclinical hypothyroidism, smoking (OR 0.766, 95% CI: 0.589-0.996, *P* = 0.047), lung cancer (OR 0.708, 95% CI: 0.567-0.884, *P* = 0.002), and esophageal cancer (OR 0.544, 95% CI: 0.360-0.821, *P* = 0.004) were protective, while hepatocellular carcinoma (OR 1.871, 95% CI: 1.438-2.433, *P* < 0.001) was a risk factor. For overt hypothyroidism, increasing age (OR 0.978, 95% CI: 0.965-0.990, *P* = 0.001) and gastric cancer (OR 0.279, 95% CI: 0.101-0.771, *P* = 0.014) were protective, whereas PD-L1 inhibitor use (OR 1.695, 95% CI: 1.017-2.825, *P* = 0.043) increased risk. For central hypothyroidism, hepatocellular carcinoma (OR 0.473, 95% CI: 0.310-0.723, *P* = 0.001) and esophageal cancer (OR 0.552, 95% CI: 0.360-0.846, *P* = 0.006) were protective, and gastric cancer (OR 1.374, 95% CI: 1.005-1.878, *P* = 0.047) was a risk factor. For thyrotoxicosis, male sex (OR 0.578, 95% CI: 0.358-0.934, *P* = 0.025) was protective, while esophageal cancer (OR 2.639, 95% CI: 1.306-5.334, *P* = 0.007) was a risk factor.

**Conclusions:**

This large real-world cohort reveals a high incidence of ICI-related thyroid dysfunction and identifies subtype-specific risk factors, underscoring the importance of routine thyroid function monitoring in high-risk patients receiving ICI therapy.

**Clinical Trial Registration:**

https://www.chictr.org.cn, identifier ChiCTR2300075974

## 1 Introduction

Immune checkpoint inhibitors (ICIs) have revolutionized cancer treatment by targeting key inhibitor pathways, including T-lymphocyte-associated protein 4 (CTLA-4), programmed cell death protein 1 (PD-1), and its ligand (PD-L1) (1–4). This blocks tumor-mediated immune evasion, revitalizes T-cell responses, and promotes tumor cell destruction (5). Over the past decade, these agents have been widely adopted in the treatment of various malignancies, significantly improving patient outcomes and becoming an important part of modern oncology practice (6).

However, while enhancing T-cell-mediated antitumor immunity, ICIs can trigger autoimmune-like manifestations involving multiple organ systems, which are commonly referred to as immune-related adverse events (irAEs) (7). Among these irAEs, ICI-related thyroid dysfunction is one of the most common endocrine toxicities. Its clinical manifestations are diverse, ranging from asymptomatic biochemical abnormalities to overt hypothyroidism and thyrotoxicosis (8). Although generally not life-threatening, these diseases can significantly impact patients’ quality of life and may require treatment interruption, thereby influencing therapeutic efficacy. Existing evidence suggests that the incidence and patterns of ICI-related thyroid dysfunction vary depending on the regimen and tumor type (9). Yet, the real-world studies are still limited to small-sample retrospective studies (10, 11). To characterize the incidence, clinical features and risk factors of ICI-related thyroid dysfunction in patients with various cancers receiving ICI therapy, this study performed a retrospective analysis of a single-center dataset. We aimed to provide actionable insights for the early detection and clinical management of these problems, thereby optimizing the safety and therapeutic efficacy of ICI-based treatment regimens.

## 2 Methods

### 2.1 Study design and data sources

This study was conducted following the amended Declaration of Helsinki and approved by the Biomedical Ethics Review Committee of West China Hospital, Sichuan University (Identifier: 2023-1064). Patients who received at least one dose of ICIs from October 2014 to June 2023 at West China Hospital, Sichuan University were included, regardless of cancer type, stage, performance status, age, or concomitant treatments. Patients with missing key data were excluded from the screening population. Among the remaining patients, those with abnormal thyroid function prior to the first ICI administration were further excluded from the final analysis. The involved individuals underwent follow-up examinations or passed away by July 4, 2025, whichever occurred first. The baseline cohort construction and data extraction from the West China Hospital big data platform have been described in our previous work on immune checkpoint inhibitor-related myocarditis (12), which shares the identical initial patient pool and enrolment window.

### 2.2 Definitions and outcomes

Based on the 2025 NCCN guidelines for Management of Immune Checkpoint Inhibitor-Related Toxicities (13), the diagnostic criteria for ICI-related thyrotoxicosis were defined as low or suppressed thyroid-stimulating hormone (TSH) with high free thyroxine (FT4) or total triiodothyronine (T3). And the ICI-related hypothyroidism was defined as follows: Subclinical hypothyroidism: normal FT4 with TSH >4.0mIU/L; Overt hypothyroidism: low FT4 with evaluated TSH; Central hypothyroidism: normal or low TSH with low FT4. The serum TSH and FT4 levels measured within 4 weeks prior to the first administration of ICIs were used as baseline values.

### 2.3 Statistical analysis

The information including gender, age, primary tumor type, laboratory test results, and other relevant data was obtained from the big data platform of West China Hospital, Sichuan University. And the details of ICI treatment as well as other medications were obtained from the electronic medical records.

All statistical analyses were performed using SPSS 29.0 and Microsoft Office 365. The baseline characteristics are presented as the mean ± standard deviation (SD) for normally distributed continuous variables, and as the median with interquartile range (IQR) for skewed continuous variables, and as the number of subjects with percentages for categorical variables. Univariate and multivariate logistic regression were performed to identify the risk factors associated with ICI-related thyroid dysfunction. Variables yielding a *P*-value < 0.20 in univariate analysis were entered into the multivariate model. Selection of predictors for the multivariate analysis was guided by clinical relevance or univariate significance. Multicollinearity was performed by calculating the variance inflation factor (VIF), thereby ensuring the independence of the explanatory variables. The findings of the multivariate analysis are expressed as adjusted odds ratios (OR) accompanied by 95% confidence intervals (95% CI). Statistical significance was defined as a *P*-value < 0.05.

## 3 Results

### 3.1 Clinical characteristics and incidence of ICI-related thyroid dysfunction

**Figure 1:**
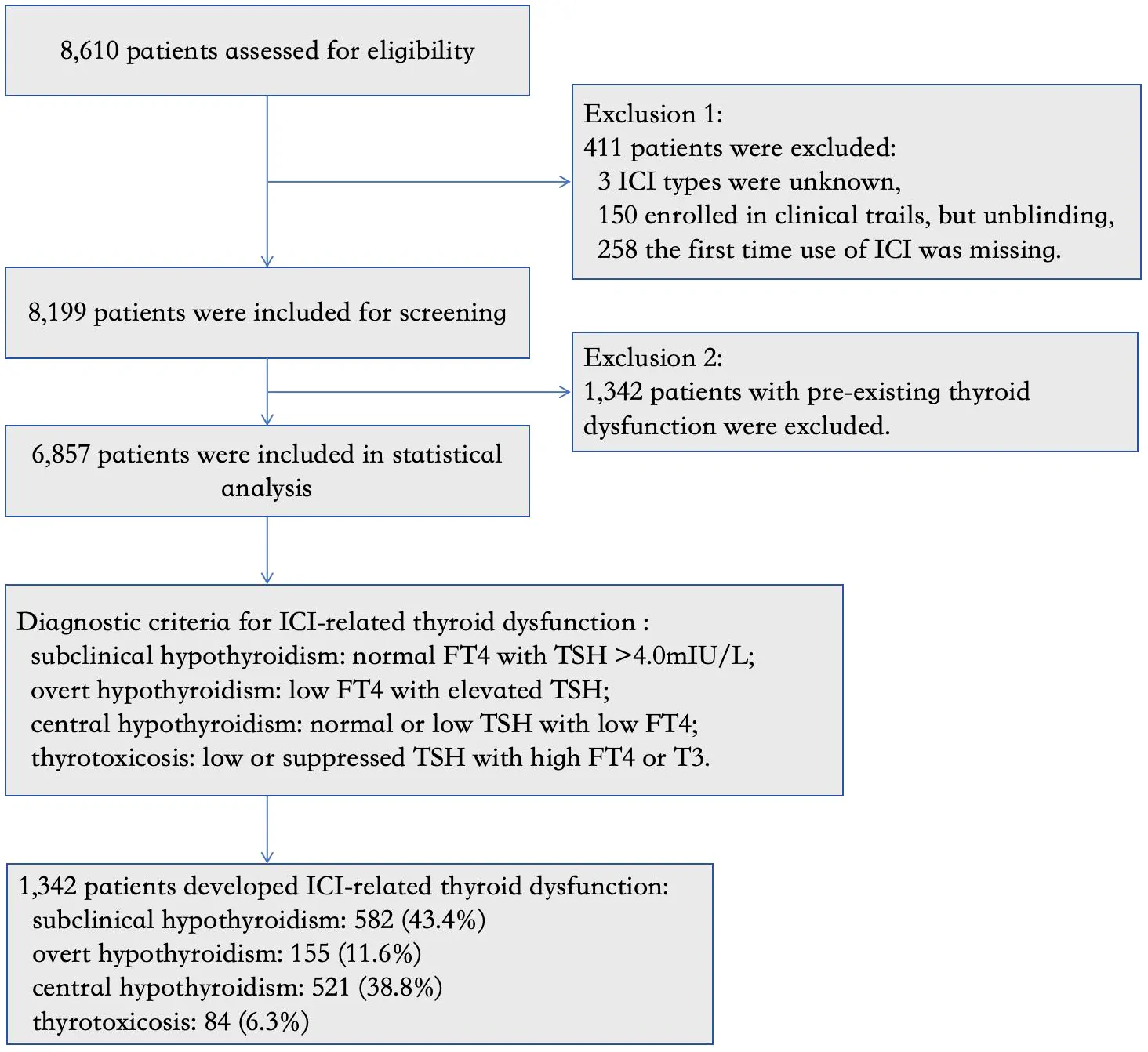
Flowchart of patients receiving immune checkpoint inhibitors: screening, enrollment, and results. The number of excluded patients with pre-existing thyroid dysfunction (n=1,342) coincidentally equals the number of incident cases (n=1,342), but these represent two entirely distinct patient populations. ICI, immune checkpoint inhibitor; TSH, thyroid-stimulating hormone; FT4, free thyroxine; T3, total triiodothyronine.

A total of 8,610 patients were initially identified. Of these, 411 patients were excluded due to missing key data, including 3 with unknown ICI types, 258 with missing timing of first ICI administration, and 150 enrolled in clinical trials but unblinding, yielding 8,199 patients included for screening. After further exclusion of 1,342 patients with pre-existing thyroid dysfunction, 6,857 patients were included in the final statistical analysis. The research process is detailed in **Figure 1**. The cohort was predominantly male (75.12%), with a median age of 58.1 years [IQR 51.7-66.6]. PD-1 inhibitors were used in 88.17% patients, and lung cancer was the most common primary malignancy, accounting for 39.32% of cases.

Among the 6,857 analyzed patients, ICI-related thyroid dysfunction was identified in 1,342 patients (19.57% overall). Specifically, subclinical hypothyroidism was observed in 582 (8.49%), overt hypothyroidism in 155 (2.26%), central hypothyroidism in 521 (7.60%), and thyrotoxicosis in 84 (1.23%). The clinical characteristics are presented in **Table 1**.

**Table 1:** Baseline demographic and clinical characteristics of ICI-related thyroid dysfunction.

Characteristic	All	Hypothyroidism			Thyrotoxicosis
		Subclinical hypothyroidism	Overt hypothyroidism	Central hypothyroidism	
Total, n	6857	582	155	521	84
Age, (years)^#^	58.1 [51.7, 66.6]	55.6 [49.9, 68.0]	54.4 ± 11.9	60.6 [50.0, 65.8]	60.6 [53.4, 66.4]
Sex					
Female	1706 (24.88)	178 (30.58)	46 (29.68)	135 (25.91)	27 (32.14)
Male	5151 (75.12)	404 (69.42)	109 (70.32)	386 (74.09)	57 (67.86)
Tobacco use, n (%)	2189 (31.92)	175 (30.07)	60 (38.71)	198 (38.00)	34 (40.48)
Alcohol use, n (%)	2027 (29.56)	143 (24.57)	43 (27.74)	141 (27.06)	27 (32.14)
Primary cancer					
Lung, n (%)	2696 (39.32)	172 (29.55)	62 (40.00)	223 (42.80)	40 (47.62)
Liver, n (%)	699 (10.19)	104 (17.87)	16 (10.32)	26 (4.99)	6 (7.14)
Gastric, n (%)	527 (7.69)	43 (7.39)	4 (2.58)	58 (11.13)	2 (2.38)
Esophageal, n (%)	575 (8.39)	28 (4.81)	5 (3.23)	26 (4.99)	13 (15.48)
Multiple primary tumors, n (%)	99 (1.44)	14 (2.41)	1 (0.65)	5 (0.96)	0 (0.00)
Other, n (%)	2261 (32.97)	221 (37.97)	67 (43.23)	183 (35.12)	23 (27.38)
Immune checkpoint targets					
PD-1, n (%)	6046 (88.17)	515 (88.49)	129 (83.23)	444 (85.22)	71 (84.52)
PD-L1, n (%)	547 (7.98)	38 (6.53)	19 (12.26)	53 (10.17)	9 (10.71)
CTLA-4, n (%)	1 (0.01)	0 (0.00)	0 (0.00)	0 (0.00)	0 (0.00)
More than one target, n (%)	263 (3.84)	29 (4.98)	7 (4.52)	24 (4.61)	4 (4.76)

^#^Age data are presented as the mean ± SD for normally distributed continuous variables, and as the median with IQR for skewed continuous variables.

IQR, interquartile ranges; PD-1, programmed cell death protein 1; PD-L1, programmed cell death ligand 1; CTLA-4, cytotoxic T-lymphocyte antigen 4.

### 3.2 Factors associated with ICI-related thyroid dysfunction

This study investigated factors associated with hypothyroidism (including subclinical, overt, and central subtypes) and thyrotoxicosis. Variables with a univariate association of *P* < 0.2 were included in the subsequent multivariate logistic regression models. Collinearity diagnostics indicated no significant multicollinearity among predictors, with all variance inflation factor (VIF) values ranged from 1.005 to 2.000, well below the conventional threshold of 5.

#### 3.2.1 ICI-related subclinical hypothyroidism

Logistic regression was first performed to identify factors associated with the development of subclinical hypothyroidism. Univariate analysis revealed significant associations between subclinical hypothyroidism and sex, tobacco use, alcohol use, primary cancer, and type of ICIs. In the multivariate model, the analysis identified smoking (OR 0.766, 95% CI: 0.589-0.996, *P* = 0.047), lung cancer (OR 0.708, 95% CI: 0.567-0.884, *P* = 0.002), and esophageal cancer (OR 0.544, 95% CI: 0.360-0.821, *P* = 0.004) as protective factors. In contrast, hepatocellular carcinoma was an independent risk factor (OR 1.871, 95% CI: 1.438-2.433, *P* < 0.001) (**Table 2**).

**Table 2:** Logistic regression analysis of risk factors for ICI-related subclinical hypothyroidism.

Characteristic	Univariate logistic regression				Multivariate logistic regression			
	*P*	OR	95%CI		*P*	OR	95%CI	
			Lower limit	Upper limit			Lower limit	Upper limit
Age	0.807	0.999	0.992	1.006				
Sex	<0.001	0.731	0.607	0.879	0.167	0.858	0.691	1.066
Tobacco use	<0.001	0.665	0.554	0.797	0.047	0.766	0.589	0.996
Alcohol use	0.006	0.759	0.623	0.925	0.951	0.992	0.757	1.299
Primary cancer								
Other	<0.001				<0.001			
Lung	<0.001	0.629	0.511	0.774	0.002	0.708	0.567	0.884
Liver	<0.001	1.613	1.256	2.072	<0.001	1.871	1.438	2.433
Gastric	0.255	0.820	0.583	1.154	0.307	0.836	0.593	1.179
Esophageal	<0.001	0.473	0.315	0.708	0.004	0.544	0.360	0.821
Multiple primary tumors	0.158	1.520	0.849	2.721	0.109	1.612	0.898	2.893
Immune checkpoint targets								
PD-1	0.280				0.384			
PD-L1	0.205	0.802	0.570	1.128	0.545	0.896	0.627	1.279
CTLA-4	1.000	0.000	0.000		1.000	0.000	0.000	
More than one target	0.157	1.331	0.896	1.978	0.112	1.384	0.927	2.066

ICI, immune checkpoint inhibitor; OR, Odds Ratio; PD-1, programmed cell death protein 1; PD-L1, programmed cell death ligand 1; CTLA-4, cytotoxic T-lymphocyte antigen 4.

#### 3.2.2 ICI-related overt hypothyroidism

Age, sex, primary cancer, and the type of ICIs were included in multivariate model. Increasing age (OR 0.978, 95% CI: 0.965-0.990, *P* = 0.001) and gastric cancer (OR 0.279, 95% CI: 0.101-0.771, *P* = 0.014) were associated with a reduced risk. Conversely, treatment with PD-L1 inhibitors was associated with an increased risk (OR 1.695, 95% CI: 1.017-2.825, *P* = 0.043) than other ICIs (**Table 3**).

**Table 3:** Logistic regression analysis of risk factors for ICI-related overt hypothyroidism.

Characteristic	Univariate logistic regression				Multivariate logistic regression			
	*P*	OR	95%CI		*P*	OR	95%CI	
			Lower limit	Upper limit			Lower limit	Upper limit
Age	<0.001	0.976	0.964	0.987	0.001	0.978	0.965	0.990
Sex	0.163	0.780	0.550	1.106	0.478	0.877	0.609	1.261
Tobacco use	0.494	0.892	0.642	1.239				
Alcohol use	0.579	0.904	0.633	1.292				
Primary cancer								
Other	0.012				0.081			
Lung	0.145	0.771	0.543	1.094	0.582	0.897	0.607	1.323
Liver	0.347	0.767	0.442	1.332	0.479	0.817	0.466	1.431
Gastric	0.007	0.250	0.091	0.690	0.014	0.279	0.101	0.771
Esophageal	0.007	0.287	0.115	0.716	0.054	0.399	0.157	1.014
Multiple primary tumors	0.279	0.334	0.046	2.432	0.395	0.421	0.058	3.084
Immune checkpoint targets								
PD-1	0.241				0.244			
PD-L1	0.045	1.651	1.011	2.694	0.043	1.695	1.017	2.825
CTLA-4	1.000	0.000	0.000		1.000	0.000	0.000	
More than one target	0.565	1.254	0.580	2.711	0.653	1.195	0.551	2.591

ICI, immune checkpoint inhibitor; OR, Odds Ratio; PD-1, programmed cell death protein 1; PD-L1, programmed cell death ligand 1; CTLA-4, cytotoxic T-lymphocyte antigen 4.

#### 3.2.3 ICI-related central hypothyroidism

According to the results of the univariate analysis, smoking, alcohol use, primary cancer, and type of ICIs were considered in the multivariate analysis. Hepatocellular carcinoma (OR 0.473, 95% CI: 0.310-0.723, *P* = 0.001) and esophageal cancer (OR 0.552, 95% CI: 0.360-0.846, *P* = 0.006) were identified as protective factors, while gastric cancer (OR 1.374, 95% CI: 1.005-1.878, *P* = 0.047) was associated with an elevated risk (**Table 4**).

**Table 4:** Logistic regression analysis of risk factors for ICI-related central hypothyroidism.

Characteristic	Univariate logistic regression				Multivariate logistic regression			
	*P*	OR	95%CI		*P*	OR	95%CI	
			Lower limit	Upper limit			Lower limit	Upper limit
Age	0.492	1.003	0.995	1.010				
Sex	0.571	0.943	0.769	1.156				
Tobacco use	0.049	0.831	0.691	0.999	0.240	0.860	0.668	1.106
Alcohol use	0.097	0.843	0.690	1.031	0.976	1.004	0.767	1.314
Primary cancer								
Other	<0.001				<0.001			
Lung	0.820	1.024	0.835	1.256	0.997	1.000	0.805	1.241
Liver	<0.001	0.439	0.288	0.668	0.001	0.473	0.310	0.723
Gastric	0.033	1.404	1.028	1.918	0.047	1.374	1.005	1.878
Esophageal	0.004	0.538	0.353	0.820	0.006	0.552	0.360	0.846
Multiple primary tumors	0.279	0.604	0.243	1.504	0.258	0.590	0.237	1.470
Immune checkpoint targets								
PD-1	0.186				0.373			
PD-L1	0.047	1.354	1.003	1.826	0.099	1.298	0.952	1.771
CTLA-4	1.000	0.000	0.000		1.000	0.000	0.000	
More than one target	0.281	1.267	0.824	1.949	0.441	1.185	0.769	1.827

ICI, immune checkpoint inhibitor; OR, Odds Ratio; PD-1, programmed cell death protein 1; PD-L1, programmed cell death ligand 1; CTLA-4, cytotoxic T-lymphocyte antigen 4.

#### 3.2.4 ICI-related thyrotoxicosis

Sex and primary cancer were finally involved in the multivariate model. Male sex was identified as a protective factor (OR 0.578, 95% CI: 0.358-0.934, *P* = 0.025), while esophageal cancer was an independent risk factor (OR 2.639, 95% CI: 1.306-5.334, *P* = 0.007). Lung cancer showed a trend to increase the risk of ICI-related toxicity, but it was not statistically significant (OR 1.672, 95% CI: 0.983-2.844, *P* = 0.058) (**Table 5**).

**Table 5:** Logistic regression analysis of risk factors for ICI-related thyrotoxicosis.

Characteristic	Univariate logistic regression				Multivariate logistic regression			
	*P*	OR	95%CI		*P*	OR	95%CI	
			Lower limit	Upper limit			Lower limit	Upper limit
Age	0.830	0.998	0.981	1.015				
Sex	0.123	0.696	0.439	1.104	0.025	0.578	0.358	0.934
Tobacco use	0.895	0.971	0.624	1.510				
Alcohol use	0.608	1.129	0.710	1.795				
Primary cancer								
Other	0.069				0.029			
Lung	0.147	1.465	0.875	2.455	0.058	1.672	0.983	2.844
Liver	0.710	0.842	0.342	2.077	0.944	0.968	0.389	2.411
Gastric	0.179	0.371	0.087	1.577	0.187	0.377	0.089	1.604
Esophageal	0.021	2.251	1.133	4.471	0.007	2.639	1.306	5.334
Multiple primary tumors	0.997	0.000	0.000		0.997	0.000	0.000	
Immune checkpoint targets								
PD-1	0.775							
PD-L1	0.338	1.408	0.700	2.832				
CTLA-4	1.000	0.000	0.000					
More than one target	0.613	1.300	0.471	3.586				

ICI, immune checkpoint inhibitor; OR, Odds Ratio; PD-1, programmed cell death protein 1; PD-L1, programmed cell death ligand 1; CTLA-4, cytotoxic T-lymphocyte antigen 4.

## 4 Discussion

ICIs have become the cornerstone of systemic therapy for multiple malignancies, and thyroid dysfunction is one of the most common adverse reactions and mostly irreversible during ICIs treatment (8, 9, 14). The clinical symptoms of hypothyroidism mainly include fatigue, cold intolerance, and bradycardia, while thyrotoxicosis typically manifests as palpitations, anxiety, tremor, and weight loss. These conditions can lead to reduced physical function, emotional distress, and may eventually compromise the quality of life in cancer patients. This large-scale, real-world study involved 6,857 participants provides a comprehensive characterization of the incidence, patterns, and risk factors for ICI-related thyroid dysfunction in a heterogeneous cohort of cancer patients treated with ICIs, offering novel insights into risk stratification and clinical management of this common irAE.

The overall incidence of ICI-related thyroid dysfunction in our cohort is 19.57%. Notably, several previous studies focusing on Chinese patients have reported relatively higher incidence. For instance, Zhang et al. (15) reported a 50.7% incidence of ICI-related thyroid dysfunction in a retrospective study of 560 cases, and Wu et al. (16) found a 44.4% rate among 270 patients. This discrepancy may be attributable to differences in sample size. Studies with relatively small sample sizes are more susceptible to selection bias and random variation, which may lead to overestimation of the true incidence and limit their generalizability.

The most common ICI-related thyroid dysfunction in our study was subclinical hypothyroidism, with an occurrence rate of 8.49%. The incidence of any type of hypothyroidism (18.35%) was significantly higher than that of thyrotoxicosis (1.23%), and 39 patients suffered from both thyrotoxicosis and hypothyroidism after receiving ICI treatment. Studies have reported that more than half of cases who suffered ICI-related thyrotoxicosis subsequently progress to hypothyroidism, with a median transition time of approximately 4 to 7 weeks (17, 18). Although the precise mechanism remains unclear, pathological analyses revealed that thyroid dysfunction associated with ICIs primarily results from the activation of cytotoxic T cells, which directly attack and destroy thyroid follicles, leading to the leakage of thyroid hormones (19). Clinical phenotypes depend on the intensity of the immune attack, and the hormone reserves of the gland. Severe follicular destruction causes transient thyrotoxicosis followed by permanent hypothyroidism, whereas mild attacks or cases with low thyroid hormone reserves may present as isolated hypothyroidism directly. Besides, PD-L1 expression has been detected on histiocytes (macrophages) accumulating within disrupted thyroid follicles and near thyroid follicular epithelial cells, suggesting that these cells may mediate local anti-inflammatory effects through PD-L1-dependent immune inhibition (20, 21). This endogenous protective mechanism may explain the finding that PD-L1 inhibitor treatment correlates with an elevated risk of overt hypothyroidism: by directly blocking tissue PD-L1, these agents eliminate this local immune checkpoint, thereby exacerbating follicular destruction and promoting sustained thyroid insufficiency.

A notable finding is the relatively high proportion of central hypothyroidism in our cohort, which accounted for 38.8% of all thyroid dysfunction cases following ICI-treatment. This difference may be explained by our standardized screening for immune-related central hypothyroidism based on criteria from NCCN guidelines, whereas prior studies mainly focused on subclinical and overt primary hypothyroidism and thus tended to underdiagnose of central hypothyroidism. Central hypothyroidism often presents with non-specific symptoms such as fatigue and lassitude, and many patients lack obvious clinical manifestations in the early stage, which further contributes to missed diagnosis in routine clinical practice. According to the latest NCCN guideline, ICI-related central hypothyroidism is diagnosed by low serum FT4 levels alongside normal or low TSH (13). Unlike other subtypes, central hypothyroidism is not caused by intrinsic damage to the thyroid gland, but by insufficient TSH stimulation of a healthy gland, which leads to defective thyroid hormone secretion (22). This defect stems from impaired upstream regulation of the hypothalamic-pituitary-thyroid axis, caused by an anatomic or functional disorder of the pituitary gland and/or the hypothalamus. Specifically, hypothalamic dysfunction can result in decreased thyrotropin-releasing hormone (TRH) production. TRH governs not only the quantity of TSH synthesis and secretion but also influences its bioactivity (23). Without adequate TRH stimulation, TSH undergoes abnormal glycosylation, resulting in a molecule that remains immunologically detectable (hence normal TSH) but lacks sufficient biological activity to stimulate FT4 secretion (24, 25). By contrast, pituitary dysfunction could lead to a reduction in TSH and FT4. Therefore, when ICI-related central hypothyroidism is suspected, comprehensive pituitary axis testing including adrenocorticotropic hormone and morning cortisol is clinically necessary to screen for concomitant ICI-related hypophysitis (26). This differential evaluation is critical to guide safe and appropriate hormone replacement. ICI-related hypophysitis frequently induces concurrent multi-hormonal deficits involving the adrenal, thyroid, and gonadal axes. Thyroid hormone accelerates systemic cortisol clearance and metabolism. If levothyroxine supplementation is initiated without prior correction of hypocortisolism, further depletion of cortisol levels can precipitate life-threatening adrenal crisis (27). Although no significant difference was identified in this study, particular attention should be paid to patients receiving CTLA-4 inhibitors, has hypophysitis has become as the most common endocrine dysfunction in this population (28). Mechanistically, CTLA-4 has been demonstrated to be expressed on anterior pituitary endocrine cells, and the binding of CTLA-4 inhibitors to these molecules activates complement-dependent cytotoxicity, which triggers anterior pituitary inflammation and destruction of endocrine cells (29).

Yet, real-world evidence on the risk factors associated with ICI- related thyroid dysfunction is still limited and inconsistent. Interestingly, smoking showed a protective effect for subclinical hypothyroidism in our cohort. Studies have found a complex relationship between cigarette smoke and thyroid function (30, 31). Evidence suggests that active or passive smoking is associated with a lower risk of subclinical hypothyroidism (32, 33). And the protective effect of smoking against thyroid autoimmunity may be attributable to its interference with iodide metabolism, suppression of pituitary TSH release, or direct immunomodulatory actions (34, 35). Furthermore, smoking has also been confirmed to be associated with enhanced immunogenicity and an activated immune microenvironment, positioning it as a potential predictor for favorable ICI treatment outcomes (36). However, the mechanism behind the protective effect of smoking remains unclear, and it is not recommended for cigarette use given its obvious detrimental impact on overall health and survival.

Moreover, in our enrolled population with a median age of 58.1 years, increasing age was found to be an independent protective factor against overt hypothyroidism (OR 0.978, *P* = 0.001). Opinions on the relationship between age and the occurrence of thyroid dysfunction are different. Consistent findings regarding the protective effect of ageing were reported in studies by Campredon et al. (37) and *Zhao* et al. (38), while *Wu* et al. found no significant connection (39). It is commonly recognized that in the general population with adequate iodine intake, advancing age is associated with an elevated risk of hypothyroidism (40), but our results suggest the impact of aging on thyroid function in patients receiving ICIs therapy may differ from that in the general population. The exact association and mechanism still require exploration through larger prospective studies. Besides, gender was found to be a significant factor contributing to ICI-related thyrotoxicosis, where female patients have a higher risk (OR 0.578, 95% CI: 0.358-0.934, *P* = 0.025). A real-world study involving 1,246 ICI-treated patients with advanced or metastatic melanoma also proofed that female gender was strongly associated with the occurrence of thyrotoxicosis (OR 2.02, 95% CI: 1.37-2.95, *P* < 0.001) (41). This predilection for women is similar to the increased susceptibility for female in other autoimmune thyroid disorders (42).

Additionally, the occurrence of different subtypes of ICI-related thyroid dysfunction may also impacted by the tumor types. In this study, lung cancer, liver cancer, and esophageal carcinoma were significantly associated with the development of ICI-related subclinical hypothyroidism. Although gastric carcinoma did not show an increased risk of subclinical hypothyroidism, it appeared to be an independent risk factor for overt hypothyroidism. Furthermore, patients with lung cancer exhibited a lower risk of ICI-related thyrotoxicosis. A precise explanation for these observed differences remains unknown. Some researchers have proposed the mechanism of cross-antigenicity or molecular mimicry (43). For example, thyroid transcription factor 1 (TTF-1) is stably expressed in both thyroid follicular epithelium and lung tissue, serving as a shared homologous protein between the lung and thyroid gland (44). A retrospective cohort study of patients with non-small cell lung cancer (NSCLC) treated with PD-1 inhibitors confirmed that tumor TTF-1 expression level was significantly correlated with the risk of ICI-related thyroid dysfunction and the clinical efficacy (45). These findings suggest that TTF-1 acts as a shared antigen mediating cross-reactivity between the lung and thyroid, enabling anti-tumor T cells to cross-attack thyroid tissue.

This study has several limitations. First, although this study benefits from a large sample size with 6,857 patients involved in the final analysis, the single-center retrospective design inherently limits the generalizability of our findings, and prospective multi-center studies are needed to validate our findings. Second, as a retrospective study, a standardized protocol for post-treatment thyroid function monitoring could not be prespecified or enforced. In routine clinical practice, the timing and frequency of TSH and FT4 testing were determined by treating physicians’ discretion, and a substantial proportion of patients may have undergone follow-up testing at outside facilities, the results of which were not captured in the institutional electronic medical record system. Consequently, the exact onset time of thyroid dysfunction could not be precisely determined, as the observed detection time reflects surveillance intensity rather than biological timing. Therefore, time-to-event analyses were not methodologically appropriate, and logistic regression was used with the outcome defined as overall detection at any time during the ICI treatment course. Third, some potential confounders, such as baseline thyroid antibody status, iodine status, concomitant medications, and prior radiation therapy, were not adjusted for in the multivariate analysis. Unlike TSH and FT4, antithyroid antibodies and iodine status are not routinely measured before the initiation of ICI therapy, as such testing is not commonly recommended by current guidelines. The relationship between antithyroid antibodies and thyroid dysfunction remains controversial. Some studies have found that thyroid dysfunction occurred more frequently in patients with positive antithyroid antibodies at baseline, particularly in those with NSCLC, melanoma, and renal cell carcinoma (46–50). While some other studies reported no significant correlation in patients treated with pembrolizumab (51, 52). Besides, cytotoxic chemotherapy alone appears to have a limited impact on thyroid function, but conventional immunomodulatory agents including interferon-α and interleukin-2 have been reported to induce autoimmune thyroid dysfunction, adding further complexity to the interpretation of concomitant medication effects in real-world settings (53). Of note, the absence of these variables in the multivariate model may have introduced bias in the observed effect estimates, and future prospective studies with standardized data collection of these potential confounders are warranted to validate and refine our findings.

## 5 Conclusion

Thyroid dysfunction is a relatively common irAEs following ICI therapy, among which subclinical hypothyroidism has the highest incidence. Smoking is a protect factor against subclinical hypothyroidism, and increasing age is associated with a lower risk of overt hypothyroidism. Female patients have higher risk for thyrotoxicosis. According to these results, it is recommended that high-risk individuals undergo more frequent monitoring of thyroid function during ICI treatment.

## Data Availability

The original contributions presented in the study are included in the article/supplementary material. Further inquiries can be directed to the corresponding author.

## Author disclaimer

This research is related to a previous study by the same authors titled ‘Clinical Characteristics and Risk Factors of Immune Checkpoint Inhibitor-Related Myocarditis: A Real World Observational Study’ (Clinical Cardiology, 2026; 49:e70312). The previous study was performed on the same single-centre cohort of ICI-treated patients at West China Hospital, Sichuan University (clinical trial registration ChiCTR2300075974;ethics 2023-1064) and analysed cardiac/myocarditis outcomes, whereas the current submission focuses on ICI-related thyroid dysfunction. The current study follows the methodology explained in that publication, which is appropriately referenced.
